# High fibrinogen and mixed proximal and distal thrombosis are associated with the risk of residual venous thrombosis in patients with posttraumatic deep vein thrombosis

**DOI:** 10.3389/fcvm.2023.1003197

**Published:** 2023-02-01

**Authors:** Yating Liu, Xiaozhi Deng, Fang Zhu, Wenhui Zhu, Zheng Wang

**Affiliations:** ^1^Department of Ultrasound, The Third Xiangya Hospital of Central South University, Changsha, Hunan, China; ^2^Central South University, Changsha, Hunan, China; ^3^Department of Vascular Surgery, The Third Xiangya Hospital of Central South University, Changsha, Hunan, China

**Keywords:** deep vein thrombosis (DVT), trauma, anticoagulation, fibrinogen (FBG), diabetes mellitus, residual venous thrombosis

## Abstract

**Background:**

The risk factors for residual venous thrombosis (RVT) in patients with post-trauma deep vein thrombosis (DVT) are unknown.

**Methods:**

We evaluated 127 patients with DVT after trauma, all of whom were treated with conventional anticoagulation and assessed for the presence of RVT with venous compression ultrasound (CUS), using an internal diameter of the venous lumen ≥ 4 mm after compression as the criterion.

**Results:**

RVT was present in 59 (46%) patients, and complete thrombus dissolution was present in 68 (54%) patients. Among them, mixed proximal and distal thrombosis (OR, 4.292; 95% CI, 1.253–14.707), diabetes (OR, 6.345; 95% CI, 1.125–35.786), fibrinogen > 4.145 g/L (OR, 2.858; 95% CI, 1.034–7.897), the time between detection of thrombus and initiation of antithrombotic therapy > 2.5 days (OR, 3.470; 95% CI, 1.085–11.094) was an independent risk factor for RVT in patients with posttraumatic DVT.

**Conclusion:**

A mixed proximal and distal thrombosis, diabetes mellitus, late initiation of antithrombotic therapy, and high fibrinogen levels increase the risk of RVT in patients with posttraumatic DVT. Therefore, treatment regimens for patients with posttraumatic DVT can be adjusted according to the site of thrombosis, the presence of diabetes mellitus, and the level of fibrinogen, and antithrombotic therapy can be started as early as possible after the detection of thrombosis to prevent the development of RVT and its serious complications.

## 1. Introduction

Trauma is the third leading global cause of death (1). Posttraumatic mortality is a three-peaked distribution, with instantaneous death, early death due to bleeding, and late death due to organ dysfunction (2, 3). With the rapid development of medical emergency technology, early to mid-term mortality after trauma is dramatically reduced. Venous thromboembolism (VTE) and pulmonary embolism are the leading causes of late trauma morbidity and mortality rates because of coagulation disorders and a hypercoagulable state of the blood (4).

Residual venous thrombosis (RVT) is a thrombus that is not entirely lysed by the fibrinolytic system and is partially organized or fibrotic after 3 months of deep vein thrombosis (DVT) occurrence (5, 6). The prolonged presence of thrombus causes persistent damage to the vascular endothelium. Studies suggest that RVT may contribute to recurrent VTE, post-thrombotic syndrome (PTS), arterial embolism, and other incidents (5–8). Other studies have shown that posttraumatic VTE is affected by factors such as age, C-reactive protein (CRP), platelets (PLT), D-dimer, and ASA levels (9). Residual thrombus after pulmonary embolism is related to no apparent inducements, acute phase fibrinogen level, and the enlarged right ventricle (10). However, one of the most pressing and unresolved issues in treating venous thrombosis is the improvement of risk stratification (5). By exploring the risk factors for RVT formation after trauma, this study will help guide clinical adjustment of treatment plans to improve long-term prognosis and avoid complications.

## 2. Materials and methods

It is a retrospective single-center study selected from trauma patients admitted to the Third Xiangya Hospital of Central South University from January 2019 to December 2021. Patients admitted for trauma with DVT (all on anticoagulation for ≥3 months and reviewed at our hospital after 3 months) were included, which is the recommended minimum duration of anticoagulation (11). Patients < 18 years old, requiring continuous hormone therapy, taking long-term anticoagulants before admission, in pregnancy or puerperium, with a history of coagulation disorders and VTE were excluded. According to the color Doppler ultrasound follow-up 3 months after discovering the thrombus, the patients were divided into the group with complete dissolution and the group with RVT. We compared the relevant data of the two groups and analyzed the independent risk factors for the formation of RVT.

Patients underwent venous compression ultrasound (CUS) within 48 h of admission to screen for DVT and received a sonographic review every 3 days during their hospitalization (12). Eventually, we enrolled 127 patients with posttraumatic DVT in the study. Antithrombotic therapy was routinely administered based on the ultrasound findings, with a review for RVT 3 months after discovering the thrombus. The diagnostic criteria for the ultrasound of RVT are that the lumen cannot be deflated when the probe is pressurized, and the width of the lumen is ≥4 mm, along with the lack of blood flow filling or no blood flow signal in the lumen (6).

The following data were collected among all patients: (1) demographic information including age, gender, body mass index (BMI), hypertension, and diabetes mellitus; (2) trauma-related factors including injury severity score (ISS), trauma location, and mechanism of injury. The mechanism of injury is divided into low-energy injuries (falls on the same plane) and high-energy injuries (falls from a height, locomotive, and vehicle injuries, hit by an object, and others). Injury severity score (ISS) ≤ 16 points to be defined as a minor injury, >16 points as a moderate injury, >25 points as a severe injury. (3) Clinical data including shock index (heart rate/systolic blood pressure), routine blood indicators (WBC, RBC, PLT, HB, CRP), coagulation function (PT, APTT, PTA, FIB, D-dimer), thrombus site [proximal (popliteal, femoral, and iliac veins), distal (calf muscle, fibular, anterior, and posterior tibial veins), and mixed proximal and distal DVT], transfusion history, and time of starting antithrombotic therapy. Laboratory indicators were obtained within 24 h of admission.

## 3. Statistical analysis

The statistical analyses were performed by SPSS version 25.0. The continuous variables that obeyed normal distribution were presented as mean ± standard deviation (mean ± SD), and the Student’s *t*-test was applied for comparison. The continuous variables that do not obey the normal distribution were presented as median (range) and compared by the Mann–Whitney U test. The continuous variables with statistical significance (*P* < 0.05) were analyzed with receiver operating characteristic (ROC) curves to calculate the optimal cut-off value and were transformed into categorical variables according to the optimal cut-off value. Pearson chi-square test or Fisher’s exact test was used to clarify the associations between each categorical variable and RVT. Logistic regression analysis was then performed, and multiple logistic regression included only those significant predictors (*p* < 0.05) in the univariate logistic regression analysis. The dominance ratio (OR) and 95% confidence interval (CI) were used to assess the degree of association between the variables and RVT.

## 4. Results

**FIGURE 1:**
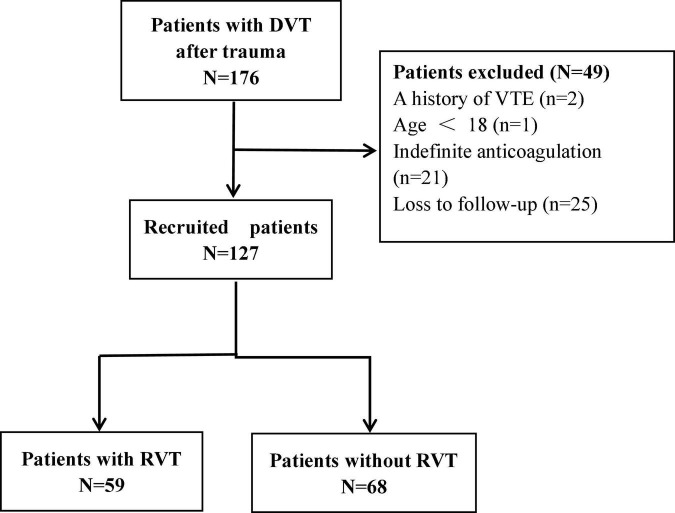
Flow diagram of the study. DVT, deep vein thrombosis; VTE, venous thromboembolism; RVT, residual venous thrombosis.

In this study, 176 patients with DVT after trauma were admitted, including 2 cases with a history of venous thromboembolism, 1 case aged <18 years, 21 cases with indefinite anticoagulation needs, and 25 cases lost to follow-up. Figure 1 shows a flow chart for selecting participants, with 127 patients eventually enrolled. The mean age of the 127 participants was 56.72 ± 15.98 years, with 81 males and 46 females. There were 25 cases of proximal thrombosis, 30 cases of mixed proximal and distal thrombosis, and the most common distal thrombosis with 72 cases. 59 (46%) of the 127 patients with remaining thrombus were in the RVT group, and 68 (54%) with complete dissolution of the thrombus were in the complete dissolution of the thrombus (NRVT) group.

### 4.1. Continuous variables and the optimum cut-off value

Table 1 compares 14 continuous variables between the RVT and NRVT groups, with statistically significant differences between the RVT and NRVT groups in fibrinogen (*P* = 0.002), time from discovery of thrombus to antithrombotic treatment (*P* = 0.012). Table 2 shows the area under the curve and the optimal cut-off values for the two continuous variables, finding an optimal cut-off value for the time between thrombosis detection and antithrombotic treatment of 2.5 days and an optimal cut-off value for fibrinogen of 4.145 g/l. Based on the cut-off values, we transformed the time from thrombus detection to antithrombotic treatment into prophylactic anticoagulation treatment, starting treatment within 2.5 days of thrombus detection, and starting treatment more than 2.5 days after thrombus detection, and transformed fibrinogen into low values (<2 g/L), normal values (2–4.145 g/L), and high values (>4.145 g/L). Thus, they were transformed into categorical variables for further Pearson chi-square test or Fisher’s exact.

**TABLE 1:** Comparison of continuous variables in patients with and without residual venous thrombosis (RVT).

Variables	Patients without RVT (*n* = 68)	Patients with RVT (*n* = 59)	*P*- value
Age (mean ± SD)	56.07 ± 15.187	57.56 ± 14.426	0.631a
BMI (median, range)	24.05 (21.58–25.63)	23.70 (22.15–26.70)	0.813b
Shock index (median, range)	0.63 (0.58–0.77)	0.63 (0.555–0.70)	0.682b
HGB (mean ± SD)	109.32 ± 26.899	107.27 ± 24.570	0.698a
RBC (median, range)	3.51 (2.82–4.19)	3.62 (2.91–4.22)	0.780b
WBC (median, range)	8.06 (6.74–10.03)	9.55 (6.61–13.075)	0.333b
PLT (median, range)	185 (135–220)	178 (136–229)	0.720b
CRP (median, range)	27.91 (12.86–77.53)	40.85 (10.09–78.59)	0.624b
PT (median, range)	12.05 (11.375–12.675)	12.20 (11.600–13.350)	0.484b
APTT (median, range)	29.05 (26.60–32.05)	27.40 (26.40–30.95)	0.259b
PTA (mean ± SD)	91.128 ± 15.222	87.104 ± 13.729	0.475a
FIB (median, range)	3.12 (2.395–3.96)	4.67 (2.78–5.67)	0.002b*
D-dimer (median, range)	3.32 (1.3775–9.6625)	6.51 (2.46–10.25)	0.103b
Time to start antithrombotic therapy (median, range)	0 (−2–1)	1 (0–3)	0.012b*

RVT, residual vein thrombosis; BMI, body mass index; HGB, hemoglobin; RBC, red blood cell; WBC, white blood cell; PLT, platelet; CRP, C-reactive protein; PT, prothrombin time; APTT, activated partial thromboplastin time; PTA, prothrombin activity; FIB, fibrinogen.

*Statistical significance.

a Student’s *t*-test.

b Mann–Whitney U test.

**TABLE 2:** The receiver operating characteristic (ROC) curve analysis of continuous variables with statistical significance.

Variable	Cut-off value	Area under the curve (95% CI)	Sensitivity	Specificity	*P*-value
FIB	4.145	0.646 (0.547–0.744)	55.9%	79.4%	0.005
Time to start antithrombotic therapy	2.5	0.631 (0.532–0.729)	42.4%	86.8%	0.012

ROC, receiver operating characteristic; CI, confidence interval; FIB, fibrinogen.

### 4.2. Univariate analysis for the categorical variables

Table 3 shows the univariate analysis of 13 categorical variables, with six factors associated with RVT: ISS (*P* = 0.001), diabetes mellitus (*P* = 0.001), hypertension (*P* = 0.046), thrombus site (*P* < 0.001), fibrinogen (*P* < 0.001), and time from detection of thrombosis to antithrombotic therapy (*P* = 0.001). Therefore, these six factors were analyzed by multiple logistic regression.

**TABLE 3:** Univariate analysis of categorical variables with interest.

Variables	Number of patients without RVT (*n* = 68)	Number of patients with RVT (*n* = 59)	*P*-value
Hypertension	22 (32.4%)	30 (50.8%)	0.046*
Diabetes	2 (2.9%)	17 (28.8%)	<0.001*
**ISS**			0.001*
≤16	55 (80.9%)	30 (50.8%)	
16–25	3 (4.4%)	10 (16.9%)	
>25	10 (14.7%)	19 (32.2%)	
**Trauma location**			0.347
Above the hip joint	6 (8.8%)	2 (3.4%)	
Hip joint and below	37 (54.4%)	30 (50.8%)	
Both	25 (36.8%)	27 (45.8%)	
**Injury mechanism**			0.459
High energy damage	47 (59.1%)	37 (62.7%)	
Low energy damage	21 (30.9%)	22 (37.2%)	
**Thrombosis location**			<0.001*
Distal thrombosis	51 (75.0%)	21 (35.6%)	
Proximal thrombosis	8 (11.8%)	17 (28.8%)	
Mixed proximal and distal thrombosis	9 (13.2%)	21 (35.6%)	
Blood transfusion	24 (35.3%)	29 (49.2%)	0.149
**FIB**			<0.001*
2–4.145 g/L	43 (63.2%)	22 (37.3%)	
<2 g/L	11 (16.2%)	4 (6.8%)	
≥4.145 g/L	14 (20.6%)	33 (55.9%)	
**Time to start antithrombotic therapy**			0.001*
≤2.5 day	33 (48.5%)	23 (39.0%)	
Prophylactic anticoagulation regime	26 (38.2%)	11 (18.6%)	
>2.5 day	9 (13.2%)	25 (42.4%)	

RVT, residual vein thrombosis; FIB, fibrinogen; reference range: 2–4 g/L, ISS, injury severity score; ISS ≤ 16 points is defined as minor injury, >16 points as a moderate injury, >25 points as a severe injury, deep vein thrombosis were classified into three types: distal thrombosis (calf muscle, fibular, anterior, and posterior tibial veins), proximal thrombosis (popliteal, femoral, and iliac veins), mixed proximal and distal thrombosis (both proximal and distal thrombosis). High energy damage (falling from a height, locomotive, and vehicle injuries, being hit by an object, and others), Low energy damage (fall on the same plane).

*Statistical significance.

### 4.3. Multiple logistic regression analysis

Table 4 exhibits the multiple logistic regression analysis of six variables, and the statistically significant factors from the univariate logistic regression were incorporated into the multiple logistic regression analysis, yielding mixed proximal and distal thrombosis (OR, 4.292; 95% CI, 1.253–14.707), having diabetes mellitus (OR, 6.345; 95% CI, 1.125–35.786), fibrinogen > 4.145 g/L (OR, 2.858; 95% CI, 1.034–7.897), the time between the detection of thrombosis to the initiation of antithrombotic therapy > 2.5 days (OR, 3.470; 95% CI, 1.085–11.094). These four factors were independent predictors of RVT.

**TABLE 4:** Multivariate logistic regression analysis of factors associated with preoperative RVT.

Variables	Univariate logistic regression analysis		Multiple logistic regression analysis	
	**OR (95% CI)**	***P*** **-value**	**OR (95% CI)**	***P*** **-value**
Hypertension	2.163 (1.053–4.444)	0.036*	1.263 (0.472–3.377)	0.642
Diabetes	13.357 (2.935–60.790)	0.001*	6.345 (1.125–35.786)	0.036*
**ISS**				
≤16	1.000			
16–25	6.111 (1.561–23.923)	0.009*	4.078 (0.599–27.786)	0.151
>25	3.483 (1.437–8.445)	0.006*	2.116 (0.448–9.991)	0.344
**Thrombosis location**				
Distal thrombosis	1.000			
Proximal thrombosis	5.161 (1.933–13.779)	0.001*	3.101 (0.617–15.585)	0.170
Mixed proximal and distal thrombosis	5.667 (2.232–14.386)	<0.001*	4.292 (1.253–14.707)	0.020*
**FIB**				
2–4.145 g/L	1.000			
<2 g/L	0.711 (0.203–2.492)	0.594		
≥4.145 g/L	4.607 (2.051–10.348)	<0.001*	2.858 (1.034–7.897)	0.043*
**Time to start antithrombotic therapy**				
≤2.5 day	1.000			
Prophylactic anticoagulation regime	0.607 (0.251–1.468)	0.268		
>2.5 day	3.986 (1.573–10.096)	0.004*	3.470 (1.085–11.094)	0.036*

*Statistical significance.

## 5. Discussion

The three main factors leading to venous thrombosis are endothelial lesions, venous stasis, and blood hypercoagulation. In trauma patients, endothelial damage and coagulation disorders caused by trauma and slow blood flow caused by braking or bed rest increased the risk of VTE (13). VTE is also a prominent contributing factor to mortality among trauma patients (14). The prolonged presence of RVT in the vessels may play an influential role in serious complications such as recurrent VTE, PTS, cancer, and atherothrombotic incidents (5–8). Repeat CUS after 3 months helps to stratify patients’ risk and guides management decisions (15). Some studies have shown that DVT is related to age, mechanism of injury, ASA score, mechanical ventilation, CRP, PLT, D-dimer, multiple injuries, prolonged hospital stay, and time from injury to surgery of more than 2 weeks (9, 16, 17). Few studies have focused on risk factors for RVT in posttraumatic DVT patients. In this study, 59 patients (46%) presented with RVT, which is consistent with previous findings of 429 RVTs detected in 869 patients (49.4%) (15). Our study showed that diabetes mellitus, mixed proximal and distal thrombosis, fibrinogen, and time from thrombus detection to antithrombotic therapy were independent risk factors for RVT after adjusting for confounding factors.

Fibrinogen, known as coagulation factor I, is an acute phase protein synthesized by the liver. It is activated into fibrin with the action of thrombin and is essential in the coagulation process of the body. Elevated fibrinogen increases blood viscosity, promotes platelet aggregation, and aggravates thrombus formation. The normal fibrinogen level in the blood is 2–4 g/L. This study revealed that acute phase fibrinogen > 4.145 g/L increases the incidence of RVT in patients with posttraumatic DVT (OR, 2.858; 95% CI, 1.034–7.897). Several studies have shown that elevated fibrinogen is related to an increased risk of venous thrombosis in a concentration-dependent manner (18–23). Furthermore, it has been found that elevated circulating fibrinogen is not only a biomarker of venous thrombosis but also has a causal relationship with the formation of venous thrombosis (24, 25).

Deep vein thrombosis is classified into proximal thrombosis (popliteal, femoral, and iliac veins), distal thrombosis (calf muscle, fibular, anterior, and posterior tibial veins), and mixed proximal and distal thrombosis according to the location of the thrombosis. It has been shown that patients with extensive deep venous system involvement are more susceptible to RVT (6). Our study also found that mixed proximal and distal thrombosis was more likely to result in RVT (OR, 4.292; 95% CI, 1.253–14.707). Compared with proximal and distal thrombosis, mixed proximal and distal thrombosis involves a wide range, seriously damages the vascular endothelium, and affects the lower extremity venous blood reflux, so entirely removing the thrombosis is difficult.

Anticoagulation in patients with DVT has been a puzzling problem to clinicians, how to minimize the danger of bleeding while allowing the complete dissolution of the thrombus (26). Then, when to start and stop anticoagulation therapy is the issue. It has been suggested that ultrasound assessment of RVT at 3 months may guide the discontinuation of anticoagulation in patients with induced DVT and that the duration of anticoagulation may be extended appropriately for those patients who have a high thrombotic risk or who develop thrombosis without apparent triggers (6). There is still no uniform standard for anticoagulation in DVT, and some scholars believe that antithrombotic therapy for distal thrombosis may increase patients’ unnecessary risk of bleeding (26–30). Hence, anticoagulation treatment of distal venous thrombosis is still an issue that needs to be explored. Our study found that starting anticoagulation more than 2.5 days after the detection of thrombus increased the risk of RVT in patients regardless of the type of thrombus (OR, 3.470; 95% CI, 1.085–11.094). We understand that delays in starting anticoagulation are usually due to active bleeding, risk of massive hemorrhage, a recent history of major surgery, and need for surgical interventions. Therefore, we recommend that anticoagulation therapy be started as early as possible for patients with thrombosis without contraindications. If patients have obvious symptoms of thrombosis or are at high risk of thrombosis, they can be given low-dose prophylactic anticoagulation therapy and then regular dose anticoagulation after the diagnosis is precise.

Diabetes is a chronic metabolic disorder. Altered intravascular homeostasis resulting from endothelial and smooth muscle cell dysfunction promotes the progress of inflammation and thrombosis (31). Although there are many inconsistent findings regarding the risk of diabetes and VTE, for patients who have developed DVT after trauma, the risk of RVT in patients with diabetes is 6.345 times higher than those without diabetes (95% CI, 1.125–35.786). It may be due to hyperglycemia-induced NO inactivation and the excessive production of reactive oxygen species (ROS), causing vascular endothelial dysfunction and inflammatory response (32). At the same time, insulin resistance increases fibrinogen and decreases tissue fibrinogen activator levels (33). Hyperinsulinemia induces monocyte tissue factor (TF) expression in diabetes patients, with a consequent increase in TF procoagulant activity and thrombin production (34). Furthermore, hyperglycemia also enhances the occurrence of these events (35). So DVT patients without diabetes are more prone to thrombus lysis.

D-dimer is a sensitive thrombotic marker commonly used for the exclusionary diagnosis of acute VTE (36). However, the sensitivity of D-dimer for thrombosis is high, but the specificity is low because D-dimer is influenced by age, inflammation, tumor, and stress response (9, 37, 38). In our study, there was no apparent difference in D-dimer between the RVT and NRVT groups, which may be because the patients were in a posttraumatic stress state and also influenced by factors such as the posttraumatic inflammatory response. In addition, we found that D-dimer was relevant to ISS in patients with posttraumatic DVT (*p* < 0.001). ISS is based on the Abbreviated Injury Scale score (AIS), which divides the body into six areas, and is the squared sum of the AIS values of the body’s three most severe injury areas (39, 40). In subsequent studies, it is promising to improve the diagnostic efficacy of posttraumatic RVT by adjusting the D-dimer cut-off value based on ISS.

Cockett syndrome, or May-thurner syndrome or iliac vein compression syndrome, is a condition in which an iliac vein is compressed by the iliac artery or has an abnormal intraluminal adhesion structure, causing obstruction of venous return in the lower extremities and pelvis, predisposing the formation of deep vein thrombosis, and making it hard to recanalize the formed thrombosis (41). Cockett syndrome is usually seen on the left side, as the left common iliac vein is compressed by the right common iliac artery anteriorly and the vertebral body posteriorly (42). The sensitivity of ultrasound for Cockett syndrome is unsatisfactory due to the interference of excessive intestinal gas, the patient’s thick subcutaneous fat layer, and the limitations of ultrasound, which affect the complete visualization of the iliac veins (43, 44). Clinicians focus the treatment of posttraumatic DVT patients on trauma repair and thrombus removal, and most patients do not improve intravascular ultrasound, computer tomography angiography, or venography. Therefore, few posttraumatic DVT patients are diagnosed with Cockett syndrome. To increase clinicians’ awareness of Cockett syndrome, we recommend a routine intravascular ultrasound, computer tomography angiography, or venography to screen for Cockett syndrome in patients with posttraumatic iliofemoral DVT, especially those with left-sided thrombosis (45). We also hope to explore the studies related to RVT by conducting multicenter studies and expanding the sample size in future studies.

## 6. Conclusion

In summary, elevated fibrinogen levels, mixed proximal and distal thrombosis, more than 2.5 days to start anticoagulation therapy, and diabetes mellitus are associated with the risk of RVT in patients with posttraumatic DVT. Risk stratification of patients with posttraumatic DVT can be performed based on factors such as D-dimer and fibrinogen levels, thrombus site, and whether or not they have diabetes mellitus to guide the adjustment of clinical treatment plans. Meanwhile, starting the antithrombotic therapy at the earliest possible time after thrombosis is detected to reduce the occurrence of RVT and its severe complications and enhance the long-term prognosis of patients.

## Data availability statement

The original contributions presented in this study are included in this article/supplementary material, further inquiries can be directed to the corresponding authors.

## Author contributions

YL designed the experiment, collected the data, analyzed and interpreted the data, and wrote the manuscript. WZ and ZW were responsible for providing the experimental ideas, guiding the experiments, and playing a pivotal role. XD collected the clinical data. FZ was in charge of reviewing and revising the manuscript. All authors contributed to the article and approved the submitted version.
